# Higher induction temperatures and the native secretion signal peptide promote rye prolamin 75k γ-secalin production in *Komagataella phaffii*

**DOI:** 10.1186/s12934-025-02809-7

**Published:** 2025-08-14

**Authors:** Kai Büchner, Christina Ludwig, Roland Kerpes, Thomas Becker

**Affiliations:** 1 TUM School of Life Sciences, Chair of Brewing and Beverage Technology, Technical University of Munich, Weihenstephaner Steig 20, 85354 Freising, Germany; 2 TUM School of Life Sciences, Bavarian Center for Biomolecular Mass Spectrometry (BayBioMS), Technical University of Munich, Gregor-Mendel-Str. 4, 85354 Freising, Germany

**Keywords:** *Komagataella phaffii*, UPR, ERAD, α-Mating factor, OST1, *Secale cereale*, Gluten, Prolamin

## Abstract

**Background:**

Gluten proteins from wheat, rye, and barley play a substantial role in human nutrition. At the same time, they can trigger several different immune reactions. This, together with their influence on the quality of grain products and their emerging role as biomaterials, makes them an interesting target for further study. The proteins’ propensity for aggregation challenges heterologous eukaryotic production systems. The yeast *Komagataella phaffii* has demonstrated excellent qualities as a production host for heterologous proteins and was therefore investigated as a platform strain.

**Results:**

A gene coding for the rye (*Secale cereale*) prolamin 75k γ-secalin was cloned and inserted into *K. phaffii*; protein expression was verified via mass spectrometry and immunoblotting and quantified via ELISA. Different parameters were investigated regarding their effect on target protein production and endoplasmic reticulum (ER) homeostasis, including the induction temperature and co- and post-translational import into the ER. At 28°C, the cells produced 1.69-fold more 75k γ-secalin than at 20°C. The introduction of the MATα-pro-region, in conjunction with either the MATα-pre- or OST1-pre-signal, led to significantly lower 75k γ-secalin accumulation, 0.20- and 0.18-fold, respectively. No mutant showed significant changes in the unfolded protein response compared to a non-producing strain.

**Conclusions:**

*K. phaffii* is a suitable host for prolamin production. The absence of a significant unfolded protein response during 75k γ-secalin expression indicates little challenge of ER-homeostasis by the aggregation-prone protein. It underscores *K. phaffii’*s imminent role in protein production. The significantly decreased protein yield through the common protein secretion leader component MATα-pro demonstrates the need for further investigation into the role of secretion signals in optimizing *K. phaffii* as a production platform for repetitive, aggregation-prone proteins.

**Supplementary Information:**

The online version contains supplementary material available at 10.1186/s12934-025-02809-7.

## Background

The storage proteins in cereal crop seed kernels, including wheat, barley, and rye, are collectively referred to as gluten proteins. They belong to the earliest described proteins [1] and are categorized into two subgroups [2]: monomeric prolamins, soluble in aqueous alcohol solutions; and polymeric glutelins, which require reducing agents to become soluble in alcohols [3]. Despite these differences, both groups share similar highly repetitive glutamine- and proline-rich regions in their primary structure [4].

Gluten proteins play a key role in the production and end product quality of grain-based foods, constituting a significant portion of the human diet [5]. Upon consumption, however, the proteins’ repetitive sequence motifs can act as immunogenic epitopes. These can cause adverse reactions such as coeliac disease [6], non-coeliac gluten sensitivity (NCGS) [7], or wheat allergy [8], which severely impact the health of susceptible individuals.

The diversity and severity of gluten protein-induced illnesses, as well as the gluten proteins’ impact on food processing and quality, render them an interesting subject of study for both medical and food scientists. In addition to their impact on health and food manufacturing, gluten proteins are also being investigated due to their potential as bio-based polymer materials [9–11]. These diverse areas of interest underscore the need for detailed studies on individual gluten proteins and their interactions in complex mixtures.

Such studies of gluten properties can be facilitated by the heterologous expression and modification of individual members of this complex protein family. Various production hosts have been employed to obtain single gluten proteins, including the prokaryote *Escherichia coli* [12–15], the yeast *Saccharomyces cerevisiae* [16–18], and insect cell systems [19]. The methylotrophic yeast *Komagataella phaffii* was proposed as a possible alternative eukaryotic host in a review article by Tamas and Shewry as early as 2006 [20]. However, to the best of the authors’ knowledge, no successful gluten protein expression in this organism has been reported to date. In general, *K. phaffii* has become a widely used protein production platform due to its ability to grow to high cell densities, its high protein secretion rate, and, compared to *S. cerevisiae*, its low tendency to hyper-mannosylate proteins [21]. One factor promoting the high protein production rate of *K. phaffii* is its constitutive base level activity of the unfolded protein response (UPR) [22]. This highly conserved response to protein-induced endoplasmic reticulum (ER) stress involves cytosolic splicing of the transcription factor HAC1^u^ (‘u’ for UPR ‘*uninduced*’) mRNA by the ER-transmembrane receptor kinase/endonuclease Ire1p to form HAC1^i^ (‘i’ for UPR ‘*induced*’) [23]. The HAC1^i^ translation product then acts as a transcription activator to alleviate ER stress, e.g. by upregulation of chaperone protein production. The ER relies on the UPR and its crosstalk with quality control systems like the endoplasmic reticulum-associated degradation (ERAD) pathways to maintain homeostasis [24]. The ERAD pathways relocate misfolded, unassembled and mistargeted proteins from the ER into the cytosol, where they are degraded by the proteasome [25]. One key protein, DOA10p, targets misfolded cytosolic and nucleoplasmic protein domains (ERAD-C [26, 27]) and ubiquitylates them to mark them for recycling [28]. Another ERAD branch uses the membrane-embedded ubiquitin ligase HRD1p, which forms a retro-translocation pore [29] to act as a part of the degradation mechanism for luminal proteins, ERAD-L [30], and substrates with misfolded transmembrane domains (ERAD-M [31]).

In plant seed kernels, gluten protein aggregation in the ER can serve as a preliminary stage for creating vacuole-like protein bodies from ER membranes in an unconventional Golgi-bypassing pathway [32]. Pronounced aggregation of wheat prolamins was also demonstrated in the *S. cerevisiae* ER [16]. However, these protein aggregates may disturb protein homeostasis and thus effect stress on the ER.

Most studies have focused on wheat-derived gluten proteins due to the high proportion of wheat in human food production. Rye is the second most commonly used grain for bread production [33], a process strongly influenced by intramolecular disulfide bond formation between gluten proteins [34]. The lack of literature concerning disulfide bond formation in rye doughs [35] makes 75k γ-secalin, with its odd number of cysteine residues [4], an interesting target for further investigation.

To the best of our knowledge, this study describes the first production of 75k γ-secalin, a prolamin encoded by the rye (*Secale cereale*) *Sec2* gene, in *K. phaffii*. The aim is to establish the yeast as a production platform to provide eukaryote-produced gluten proteins for future research, and leverage its advantageous characteristics, such as efficient protein expression, cost-effectiveness, and the well established manipulation of post-translational protein processing [36]. Different strategies for production optimization, i.e. a lowered temperature during methanol induction and the use of different secretion signals, were compared regarding protein production quantity. Additionally, the level of ER stress exerted by the protein in *K. phaffii* was analyzed with HAC1 mRNA splice variants as markers.

## Results and discussion

### Strain construction

The *Sec2* gene was PCR-amplified with a high-fidelity polymerase directly from genomic *S. cereale* cv. Elias DNA, since sequence data retrieved from the NCBI database revealed no introns in the 75k γ-secalin coding region. These sequence data were also used to generate a consensus sequence, which served as a template for the design of primers annealing at the genes initiation and termination codons. *K. phaffii* cells were transformed with a linear construct containing a phleomycin D1 (Zeocin™) resistance gene and the *S. cereale* *Sec2* gene under the control of the methanol-inducible CAT1 promoter [37]. The *Sec2* coding region of positive transformants was then sequenced (GenBank accession number PQ843236), and a 75k γ-secalin expressing strain was verified through immunoblotting and termed *K. phaffii* Z-gSec.

**Fig. 1:**
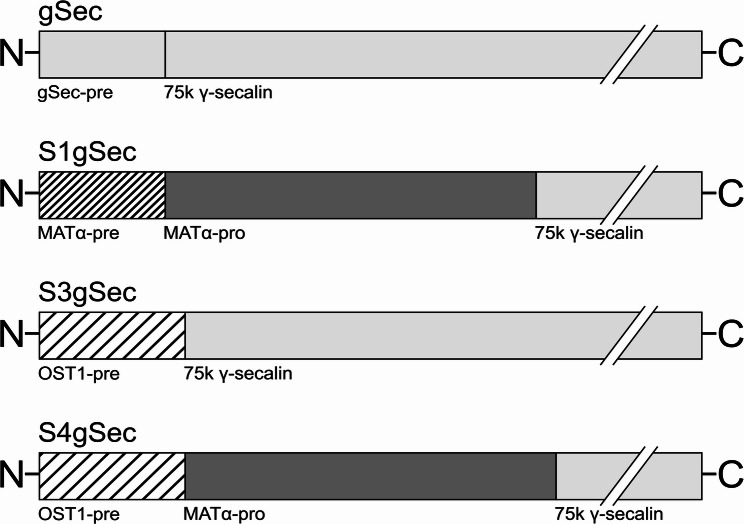
Schematic representation of the signal sequence variants for 75k γ-secalin. The *in silico* predicted native 75k γ-secalin-pre-peptide is designated gSec-pre, whereas the light gray 75k γ-secalin represents the complete, mature protein

Mutations were introduced into the *Sec2* gene in *K. phaffii* Z-gSec by homologous recombination to avoid metabolic effects of the expression cassette integrating into random genomic sites. The strain was transformed with three separate gene cassettes, each carrying a geneticin resistance gene as well as a replacement for the native signal sequence, either i) the *S. cerevisiae* α-mating factor prepro-peptide (MATα) (S1gSec), ii) the OST1-pre-signal (S3gSec), or iii) the OST1-pre-MATα-pro-signal (S4gSec) (Fig. 1). Clones were screened for successful integration via replica plating on 0.1 mg/mL phleomycin D1 and 1.0 mg/mL G418 in YPD plates, respectively. Positive candidate strains grew on media containing G418 but not on phleomycin D1 media. The integration of the mutations was confirmed by sequencing; one clone, carrying the G418 resistance gene but no mutations to the *S. cereale* *Sec2* gene, was termed *K. phaffii* gSec and kept as the native strain for comparative analyses. The same strategy was applied to generate a *Sec2* null mutant, where the first 79 base pairs (bp) of the 5’ region were removed to delete any in-frame start codons, and a single nucleotide deletion was introduced at bp position 92 of the native gene (bp position 12 of the null mutant gene) as a redundant frameshift safeguard against translation. The *Sec2* null mutant was termed *K. phaffii* ΔgSec and used as a reference strain for further RT-qPCR experiments.

### 75k γ-secalin *in silico* analysis reveals immunogenic epitopes

**Fig. 2:**
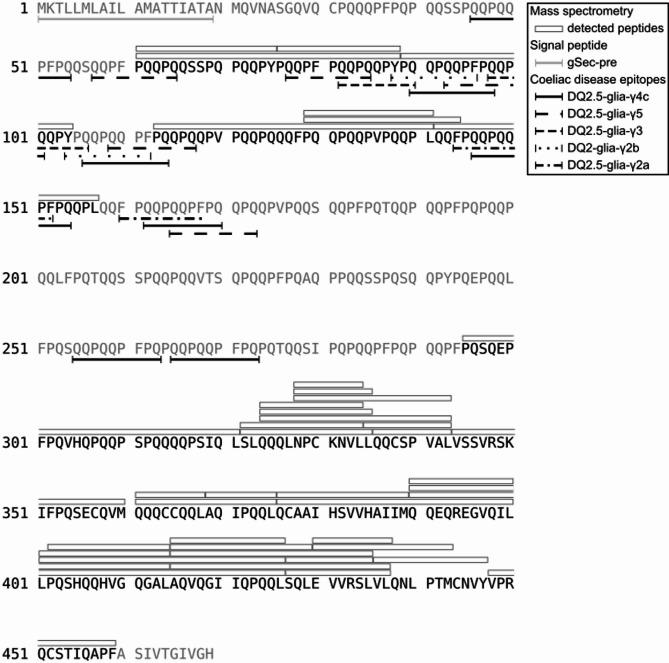
Translation of the *S. cereale* cv. Elias *Sec2* gene. The protein sequence was translated *in silico* and is shown in gray, the parts confirmed by mass spectrometry are shown in black. White boxes above the sequence denote the identified peptides. Amino acid positions 1 to 19 show the native secretion signal (underlined in gray). Five common coeliac disease epitopes [38] were found in the rye secalin, and are underlined in different dashed lines (see legend)

The prolamin 75k γ-secalin, produced from the *Sec2* gene of rye cultivar Elias, was predicted to consist of 469 amino acid (AA) residues with a native signal peptide of 19 AA. Figure 2 shows the *in silico* translation product and signal sequence, as well as the peptide coverage detected by mass spectrometry. The protein’s molecular mass was calculated to be 53315.12 Da with and 51354.31 Da without the native signal sequence. A sequence search revealed 19 coeliac disease epitopes also found in wheat [38] (Fig. 2). In general, coeliac disease epitopes are shared among gluten proteins from wheat, barley, and rye [39], highlighting the importance of the analysis of non-wheat cereal grains regarding potential health impacts: analyzing IgE binding specificity to epitope sequences requires well-characterized proteins as it depends on protein sequence, linear epitopes, and structure, which affects epitope presentation and activation. Protein folding, influenced by disulfide bridges, is crucial for this conformation [40]. The 75k γ-secalin produced in this study contains nine cysteine residues, an odd number suggesting disulfide bridges beyond intramolecular bonds [3, 4], with a potential impact on the protein aggregation behavior in the ER and other organelles.

### Protein mass spectrometry confirms 75k γ-secalin production in K. phaffii gSec

Three biological replicates of *K. phaffii* gSec and one sample of *K. phaffii* ΔgSec fermentations at 28°C were investigated via mass spectrometry to confirm 75k γ-secalin production on the protein level. Isopropanol-extracted samples were separated on a polyacrylamide gel, and a protein band at a molecular weight of approximately 72 kDa was excised, corresponding to bands visualized through immunoblotting, as discussed below. Proteins were digested with chymotrypsin and measured on a high mass accuracy and high-resolution mass spectrometer. The resulting mass spectra were searched against the complete *K. phaffii* proteome appended with the amino acid sequence of rye 75k γ-secalin (Sec2p). In total, 41 Sec2p-specific peptides were detected, covering 54% of the protein sequence (Fig. 2). The peptides cluster in three regions, positions 61 to 104, positions 113 to 157, and positions 295 to 459, leaving the N-terminus and a 137 AA repetitive middle sequence of the protein uncovered. Due to eight chymotrypsin cleavage sites within the first 21 N-terminal AA, a lack of sequence coverage of the native secretion signal (position 1 to 19) was expected. The middle part of the protein, rich in glutamine and proline, does not contain cleavage sites for chymotrypsin. Therefore, the resulting peptide was likely not small enough for mass spectrometry analysis. Eight of the nine predicted cysteine residues are located in the C-terminal third of the protein and can be found in the covered sequence. None of the peptides were found in the *K. phaffii* ΔgSec strain, confirming the strain as a null mutant.

### A high fermentation temperature enhances 75k γ-secalin production

**Fig. 3:**
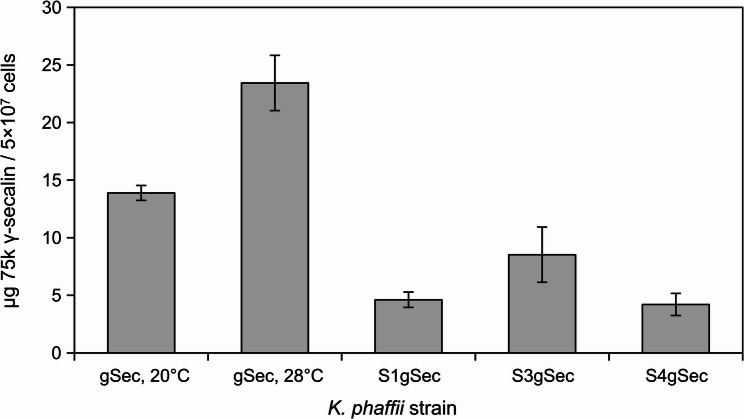
ELISA quantification of 75k γ-secalin produced in *K. phaffii*, normalized to 5 × 10^7^cells. The strains are designated according to fed-batch temperature (both with the native pre-factor, *n* = 3 each) and secretion signal mutation (S1gSec, MATα-prepro; S3gSec, OST1-pre; S4gSec, OST1-pre-MATα-pro; all fed batches at 28°C, *n* = 3)

**Fig. 4:**
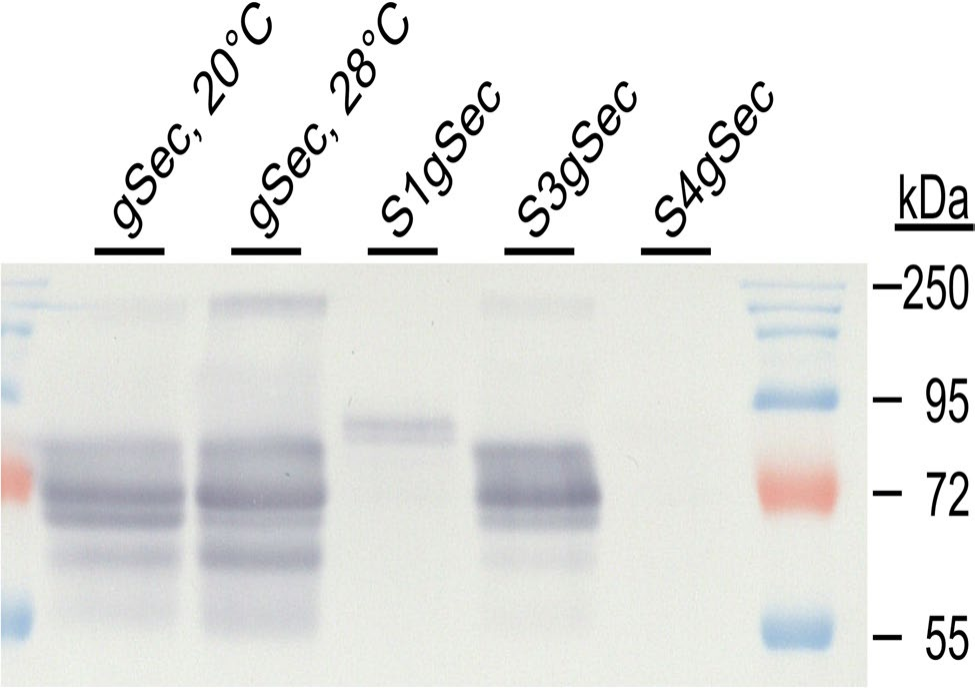
Immunoblot of propan-2-ol extracted cell lysates of 75k γ-secalin expression strains. Electrophoresis was performed using the NEB pre-stained protein ladder 10–180 kDA in a 10% tris-glycine gel

While lower temperatures during *K. phaffii* induction increase the expression of heterologous proteins [43, 44], higher fermentation temperatures increase the aggregation and retention of such proteins in the ER [45]. Here, the methanol fed-batch phase was conducted at 20°C and 28°C each with the strains *K. phaffii* gSec and ΔgSec to investigate temperature influence. ELISA-measurements showed a 75k γ-secalin yield Y_P/X, 20°C_ = 13.89 ± 0.65 µg/5 × 10^7^ cells for cultivations at 20°C and Y_P/X, 28°C_ = 23.43 ± 2.39 µg/5 × 10^7^ cells for 28°C fed-batch cultivations (Fig. 3). No 75k γ-secalin was detected in the supernatant of the cultures (data not shown), suggesting the intracellular accumulation of the target protein. Immunoblotting with an anti-gliadin antibody revealed a similar pattern of six bands (56 kDa, 63 kDa, 67 kDa, 71 kDa, 81 kDa, and 185 kDa) for both fermentation temperatures, while individual band intensities differed (Fig. 4). Negative controls extracted from ΔgSec 28°C-fermentations did not show any bands in the immunoblot (data not shown). The band at 71 kDa was most intense at both induction temperatures. This relative mass contradicts the predicted protein size of 53.3 kDa but is consistent with the literature, where secalins run in the 75 kDa size range on SDS-PAGE despite their predicted size of 53 kDa [46]. Current theory proposes that this is due to the protein’s aggregation and the low resolution of SDS-PAGE analyses [47]. Also, the Tris-Glycine buffer system itself might play a role in the anomalous protein migration [48], but for compatibility with the downstream mass-spectrometry protocols, the established Laemmli protocol was followed. Specifically in *K. phaffii*, where the trafficking routes of gluten proteins are not yet established, a plausible reason for the higher apparent protein weight might be found in covalently bound post-translational modifications. One example of such post-translational modifications, O-glycosylation in the Golgi apparatus, targets serine and threonine [49], which can be found 30 and 11 times in the 75k γ-secalin expressed here, respectively, and therefore make it a modification likely to occur. The unusual behavior of gluten proteins on Laemmli SDS gels highlights the necessity for cautious interpretation of the other bands revealed by immunoblotting. The higher intensity of the 67 kDa band at 20°C indicates shortening of the 71 kDa protein and a higher rate of protein-processing than at 28°C [45]. Removal of the native 19 AA secretion signal motif (approx. mass: 2 kDa) might account for the relative mass difference. Recent literature suggests a wide variety of crosslinks between prolamine monomers depending on the solvent [50]. Therefore, the bands migrating at 81 kDa and 185 kDa might be formed by 75k γ-secalin oligomers. The 63 kDa band, which differed from the native protein by 8 kDa, is more pronounced in the 28°C sample. The existence of a so far undescribed prolamin pro-peptide might explain this mass difference, but iterative *in silico* mass calculations of fragments starting at the N-terminus remain inconclusive: the mass difference is most closely approximated through amino acids 1 to 73. However, a considerable part of this peptide consists of repetitive motifs that start at AA position 33. Since there are no distinguishing features between the sequence from 33 to 73 and the following repetitive motifs, the processing of such a hypothetical pro-region remains questionable. Due to the many possible reasons for their occurrence and the possible insights into gluten protein trafficking associated with it, the nature of the several distinct gluten protein masses produced by *K. phaffii* gSec requires further investigation.

The stronger 67 kDa band indicates a more efficient 75k γ-secalin processing in the ER at a lower induction temperature, but since the higher fed-batch temperature significantly increased the production of the rye prolamin (*p* = 0.045), 28°C was adopted as the induction temperature for all further experiments.

### ACT1 and TFC1 are the most stable RT-qPCR reference genes

The most stable set of reference genes for RT-qPCR relative quantification was determined with mRNA samples from *K. phaffii* ΔgSec fermented at 20°C and *K. phaffii* gSec fermented at 28°C. This allowed for the selection of constitutively expressed genes independent of growth temperature and 75k γ-secalin production. Candidate genes were selected from literature (ACT1, TDH3, and TPI1 [51]; ALG9, TAF10, and TFC1 [52]; ENO1, PDA1, and PFK1 [53]), considering minimized crosstalk between the regulation of individual genes. The geNorm algorithm used for reference gene analysis [54] revealed the most stable expression for ACT1, TFC1, and ENO1 (supplementary material 1). ACT1 codes for the cytoskeletal protein actin and is widely used in the *K. phaffii* research community, although its perpetual expression stability is disputed [55]. TFC1 codes for a subunit of the RNA polymerase III transcription initiation factor complex [52], whereas ENO1 encodes enolase I, which is involved in glycolysis and gluconeogenesis [56]. Since the geNorm V-values lay below 0.150 for all combinations of numbers of genes (supplementary material 1), ACT1 and TFC1 were used as a reference gene system for all RT-qPCR relative quantification experiments in this study.

### 75k γ-secalin production does not challenge K. phaffii ER-homeostasis

After 96 h of methanol fed-batch fermentation, gene expression was studied in *K. phaffii* gSec relative to *K. phaffii* ΔgSec in both 20°C and 28°C fed-batch cultures. RT-qPCR analysis targeted the splice-variants of transcription factor HAC1 mRNA, HAC1^u^ and HAC1^i^, and the genes DOA10 and HRD1, which are involved in different endoplasmic reticulum-associated degradation (ERAD) pathways. The unfolded protein response (UPR) transcription factor HAC1 and its homologs are common among eukaryotic organisms (reviewed in [57, 58]). HAC1 is constitutively transcribed to its uninduced form, HAC1^u^, which is spliced in response to protein-generated ER stress to create the induced form HAC1^i^. This atypical cytosolic splicing is performed by ER-resident transmembrane kinase/ribonuclease (RNase) inositol-requiring enzyme 1 (IRE1) [59], which is activated by accumulation of aberrant protein in the ER-lumen [60].

**Fig. 5:**
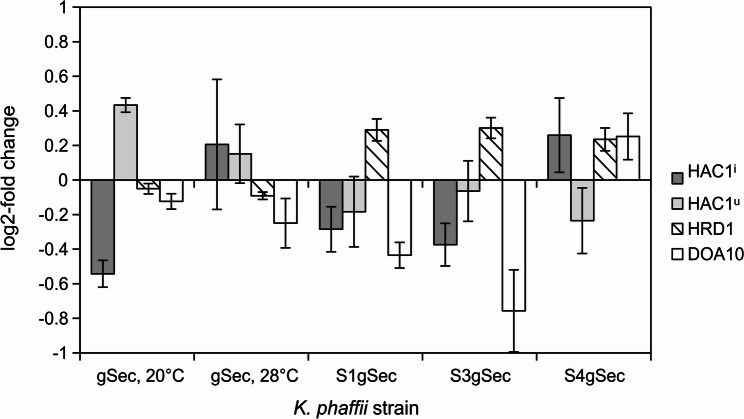
Relative quantification of mRNA. Expression of the genes of interest (GOIs) in *K. phaffii* gSec at 20°C is shown relative to expression in *K. phaffii* ΔgSec at 20°C. The GOI expression in *K. phaffii* gSec 28°C, S1gSec, S3gSec, and S4gSec is shown relative to K. phaffii ΔgSec at 28°C. All samples were harvested after a 96 h methanol fed-batch phase at the respective temperatures

Prolamins expressed in *S. cerevisiae* aggregated in the ER to form dense protein bodies [16]. In plant seed cells, this behavior triggers a specific transport pathway leading to ER-derived protein bodies [32], but even in native hosts, current literature suggests a challenge of the ER’s protein processing machinery through gluten protein aggregation [61]. The increased intracellular accumulation of native 75k γ-secalin in *K. phaffii* gSec at 28°C is concomitant with a small, not significant, increase (1.32-fold) in HAC1^i^ abundance compared to the non-producing *K. phaffii* ΔgSec strain (Fig. 5). Furthermore, due to differing growth behavior and relative gene expression, one flask of the *K. phaffii* gSec triplicate at 28°C was excluded from the analysis. At 20°C, the behavior of the HAC1 mRNA is remarkably different from that at 28°C: the relative abundance of HAC1^i^ is reduced to 0.69-fold, while HAC1^u^ abundance remains stable at the 1.02-fold of the null mutant. Despite the ELISA showing target proteins only in the lysed cell fraction and therefore suggesting intracellular protein accumulation, the HAC1^i^ abundance is not significantly heightened; additionally, the ERAD-associated genes DOA10 and HRD1 are stably expressed at either temperature. In conclusion, neither the ERAD pathways nor the UPR were significantly upregulated after 96 h of rye prolamin expression. These results support *K. phaffii*’s role as a widespread protein production platform since a protein that demonstrated high aggregation propensity was produced without posing a significant challenge to ER homeostasis.

### MATα-pro-region has an adverse effect on 75k γ-secalin production

The native signal peptide of 75k γ-secalin was predicted *in silico* and replaced by three different signal sequences to investigate the influence of diverse signal peptides on the processing of the proteins. Literature research did not indicate any potential pro-peptides for rye prolamin; the hydropathy distribution of the native protein’s primary structure [62] also did not reveal any hydrophobic motif outside the predicted signal sequence that could indicate a pro-peptide, as described above. As no clear pro-peptide could be identified, only the predicted 19 AA signal sequence was replaced by MATα-prepro, OST1-pre, and OST1-pre-MATα-pro. The *S. cerevisiae* MATα-prepro signal is widely used in *K. phaffii* expression systems for post-translational import into the ER and subsequent protein trafficking through the secretory pathway [63]. The OST1-pre-signal promotes co-translational import into the ER [64]. The third alternative signal sequence, a chimera of OST1-pre and MATα-pro [65], was shown to enhance secretion through its pre-peptide and reduce protein aggregation through its pro-peptide [64]. Upon ER import, the chaperone KAR2p acts as a Brownian ratchet that furthers the forward movement of nascent polypeptides into the ER [66]. It has been suggested that MATα-pro may stimulate translocation across the ER-membrane by providing a long stretch of unfolded polypeptide upstream of the folded passenger protein, allowing Kar2 to act more effectively [67]. Both mutant strains containing the MATα-pro-region, *K. phaffii* S1gSec and S4gSec, divert considerably from the production behavior of the native 75k γ-secalin-producing strain (Fig. 4). ELISA quantification of prolamin extracted from cell-lysates showed significantly, almost five-fold lower accumulation than the native strain (Y_P/X, S1gSec_ = 4.61 ± 0.67 µg/5 × 10^7^ cells, *p* = 0.005; Y_P/X, S4gSec_ = 4.20 ± 0.96 µg/5 × 10^7^ cells, *p* = 0.005). None of the bands associated with native 75k γ-secalin were present in the immunoblot. Instead, the lysate from strain S1gSec displayed two bands with relative masses of 83 kDa and 87 kDa, respectively. These changes in size compared to the native core protein (approx. 67 kDa, excluding the native secretion leader) are explained by the presence of the MATα-prepro- (87 kDa) and the MATα-pro- (83 kDa) region in the engineered strain. The presence of both protein variants also indicates the removal of the MATα-pre-region and, thus, an active processing of the protein in the ER. The size of MATα-pre was calculated to be 2 kDa, which would account for half of the mass difference between proteins shown by SDS-PAGE separation. This consistency hints at an overestimation of the secretion leader mass by gel electrophoresis, which might be due to the mobility characteristics of the gluten protein itself. Similarly, the relative mass of the MATα-pro-peptide was calculated to be 6 kDa, with a size overestimation possibly causing the 16 kDa size difference to the native core peptide. Beyond the two bands described, no bands of other sizes were found, showing no visible indication of further processing or degradation stages of 75k γ-secalin. Despite producing a similar amount of gluten protein according to ELISA measurements, the S4gSec sample showed no bands on the immunoblot (Fig. 4). As the R5 antibody used in the ELISA kit is able to detect decapeptides [68], it stands to reason that the rye prolamin with the chimeric secretion signal was expressed by *K. phaffii* S4gSec, but experienced degradation within the cell. This would result in smaller peptides that are not detected by SDS-PAGE separation but rather by ELISA. *K. phaffii* S3gSec, although producing significantly lower amounts of 75k γ-secalin (*p* = 0.012), shows approximately twice the protein yield of the MATα-pro-mutants (Y_P/X, S3gSec_ = 8.52 ± 2.39 µg/5 × 10^7^ cells). Immunoblotting revealed a band pattern similar to that of the native strains, as the band at 71 kDa is the most intense. The other bands between 67 kDa and 81 kDa are less distinct, while the bands at 56 kDa, 61 kDa, and 185 kDa are comparatively faint. This indicates a higher turnover and degradation through the co-translational OST1-pre-signal. Furthermore, lower yields seem to be concomitant with the MATα-pro-region. *In silico* structure prediction [69] showed five α-helices in the N-termini of all secretion signal variants. Therefore, neither the co- or post-translational ER-import nor the structural properties of the different secretion signals provide any indication of the different behavior of the MATα-pro-mutants. Interestingly, the MATα-pro-region shows one N-glycosylation motif at position four (-N-T-T-). In contrast, 75k γ-secalin from rye cultivar Elias contains no N-glycosylation motifs. In the ER-lumen, glycans are attached to the asparagine residue of the N-glycosylation motif -N-X-S/T- to serve as protein processing signals [70]. The influence of N-glycosylation on protein processing in *K. phaffii* has been demonstrated previously [71], so the influence of artificial N-glycosylation on the ER-processing of natively non-glycosylated proteins (such as 75k γ-secalin) requires further investigation.

### Changes in ERAD activity are associated with secretion signal modifications

Compared with the null mutants, the secretion signal mutants S1gSec, S3gSec, and S4gSec generally do not significantly (*p* = 0.05) differ in the abundance of HAC1^i^ and HAC1^u^ or in HRD1 and DOA10 expression. In *S. cerevisiae*, the expression of γ-gliadin, a γ-secalin-equivalent wheat prolamin, led to the formation of dense protein bodies in the ER [16]. This challenge to ER homeostasis can be expected to be answered by HAC1 induction through splicing and, via a feedback loop, a higher total HAC1 transcription that increases HAC1^u^ abundance. Wheat gliadins aggregate at physiological pH [72]. Hence, the yeast cytosolic pH of 6.8 and ER pH of 7.2 [73] should support aggregation and hinder ER import, making co-translational signals more effective due to their translational pause [74]. However, the lack of significant differences between the UPR intensity in the four signal sequence variant strains suggests no difference in 75k γ-secalin protein aggregation with co- and post-translational signal sequences. The uniform behavior of all strains, independent of ER import mode, was unexpected due to disulfide bond formation: the ER lumen is an oxidizing environment compared to the cytosol [30] and thus promotes the formation of disulfide bonds [75]. The odd number of cysteine residues in the 75k γ-secalin produced in this study might render it prone to form intermolecular disulfide bridges that further protein agglutination. Co-translational ER-import prevents protein aggregation through efficient chaperone recruitment [76] and circumvents highly aggregation-prone non-native intermediates caused by non-native disulfide bonds [77]. However, the low UPR-activation measured through HAC1^i^-abundance in all strains indicates little, if any, prolamin aggregation in the ER. Together with the similar band patterns of *K. phaffii* strains gSec and S3gSec, and the low protein accumulation in MATα-pro-strains S1gSec and S4gSec, the low stress response shown in the RT-qPCR data suggests that processing of 75k γ-secalin by *K. phaffii* depends more on the secretion leader pro-signal than on the ER-import mode. This is most likely to the basal UPR activity in the native strain [22], which renders *K. phaffii* an exceptional protein production host.

Notably, while all secretion signal mutants display a higher relative HRD1 expression than the native *K. phaffii* gSec strain (p_S1_= 0.059, p_S3_ = 0.056, p_S4_ = 0.071), only the chimeric OST1-pre-MATα-pro-signal mutant S4gSec shows a heightened DOA10 expression (p_S4_ = 0.069). In *S. cerevisiae*, DOA10 is regulated by four genes (GCN4, SFP1, XBP1, and YAP5; see [78]). Only SFP1 and GCN4 have been annotated in *K. phaffii* [79]. Both genes were shown to be influenced by neither the overexpression of *S. cerevisiae* HAC1 nor the addition of dithiothreitol (DTT), a UPR-inducing stimulus; additionally, DOA10 expression was not influenced by either condition [80]. This leads to the conclusion that the DOA10-dependent ERAD branches are upregulated separately from the UPR, further supported by increased DOA10 expression with simultaneously non-significant UPR-activation in the strain S4gSec, where cytosolic 75k γ-secalin degradation can be assumed due to the lack of bands in the immunoblots. The comparatively lower influence of 75k γ-secalin production on HRD1 expression suggests the involvement of the ERAD-L and -M pathways in all secretion signal mutants, although no bands were visible in an anti-ubiquitin immunoblot (data not shown).

## Conclusions

This study focused on the expression of the *S. cereale* prolamin 75k γ-secalin in *K. phaffii* and strategies to optimize its expression with respect to yield and cell stress. The target gene was cloned from commercial rye cultivar Elias, and its *in silico*-predicted sequence was verified via mass spectrometry. The highest protein yields were achieved at 28°C with the native protein, while 20°C led to lower protein yields, albeit with more efficient processing of the native signal sequence. Introduction of the MATα-pro-region together with both co- and post-translational signal sequences significantly decreased 75k γ-secalin accumulation in the cell. The high efficiency of *K. phaffii* as a production platform was demonstrated by only minor increases in UPR activity as a proxy for ER-stress in all tested strains. Instead, all non-native secretion signals increased HRD1 transcription, suggesting the involvement of the ERAD-L and -M pathways in 75k γ-secalin degradation. Furthermore, the combination of the MATα-pro-region with the co-translational OST1-pre-signal seems to activate the ERAD-C pathway, shown by increased DOA10 transcription. The similarity of the *in silico*-predicted secondary structures of all signal sequences, native and mutant, does not explain the differing behavior of the non-native secretion signals. Furthermore, the apparent superiority of the native rye secalin secretion signal for intracellular protein production, its influence on protein half-life, and the underlying mechanisms pose interesting subjects for further investigations.

## Methods and materials

### Strain construction

*Komagataella phaffii* BSY BG11 strains with mut^S^ phenotype and all plasmids were obtained from Bisy GmbH, Austria. Mutant strains were selected and grown at 28°C and 180 rpm in baffled shaker flasks with rich medium (YPD, 2% w/v peptone, 1% w/v yeast extract, 2% w/v glucose) supplemented with either phleomycin D1 (Zeocin™, 100 µg/mL) or G418 (1 mg/mL) depending on the integrated plasmid. Genomic DNA was isolated from *S. cereale* cv. Elias seeds using the CTAB method [81]. The *Sec2* gene coding region was amplified with ALLin™ Mega HS HiFi DNA Polymerase (HiQu, Germany) and primers gSEC-FWD and gSEC-REV according to the manufacturer’s instructions (for primer sequences, see supplementary material 2). All further amplifications were performed with NEB Q5 polymerase according to the manufacturer’s instructions (New England Biolabs GmbH, Germany). The Sect. 2 amplicon was merged with the upstream (pBSYleft-FWD, pBSYleft-REV) and downstream (pBSYright-FWD, pBSYright-REV) elements of the vector pBSY3Z by overlap extension (OE-) PCR. The linear cassette was introduced into *K. phaffii* BSY BG11 cells through electro-transfection [82]. Transformants selected on YPD plates with phleomycin D1 were then incubated in BMM1 (1.34% (w/v) YNB, 0.5% (v/v) methanol, 4 × 10 − 5% (w/v) biotin, 200 mM potassium phosphate buffer pH 6.0) and screened for 75k γ-secalin production by immunoblotting of propan-2-ol + DTT extracts of lysed cells as described below. One production strain was selected and termed BSY3Z-gSec. The phleomycin D1 resistance gene of the original vector was replaced by the KanMX gene via OE-PCR (pBSYleft-FWD, KanMX-BSY-REV; KanMX-FWD, KanMX-REV; KanMX-BSY-FWD, pBSYright-REV), to create cassette BSY3G-gSec. The expression cassette was additionally modified through OE-PCR with the upstream portion of the plasmids pBSY3S1Z (pBSYleft-FWD, proMATα-gSEC-REV), pBSY3S3Z (pBSYleft-FWD, preOST1-gSEC-REV), and pBSY3S4Z (pBSYleft-FWD, proMATα-gSEC-REV). The right-hand side was amplified from the BSY3G-gSec cassette with primers mature_gSEC-FWD and pBSYright-REV. The *Sec2* null mutant cassette BSY3G-ΔgSec was constructed using OE-PCR with a left-hand side (pBSYleft-FWD, D-gSEC-REV) and a right-hand side (D-gSEC-FWD, pBSYright-REV) amplified from the BSY3G-gSec template.

Transformants were grown at 28°C on YPD G418 plates and then replica-plated on YPD phleomycin D1 plates. Cultures growing in the presence of G418, but not phleomycin D1, were selected. Sequence identity and correct integration of the cassettes were verified by PCR and Sanger sequencing (Eurofins Genomics Europe Shared Services GmbH, Germany) with primers pBSYseq2-FWD and pBSYseq-REV. Sequence data were analyzed using the NCBI BLAST [83], *in silico* translations of the gene sequence, and analysis of protein parameters were performed on the ExPASy server [84]. A protein signal sequence was predicted with SignalP 6.0 [85].

### Extraction of yeast genomic DNA

Genomic DNA was extracted from *K. phaffii* cells according to [86], modified according to [87]. In brief, a cell pellet was resuspended in lysis buffer (0.2 M lithium acetate, 1% (w/v) SDS) and incubated at 75°C for 10 min. Three volumes of absolute ethanol were added, the solution was spun down, and the pellet was washed twice. The pellet was resuspended in nuclease-free water, debris was spun down, and the supernatant was used as PCR template.

### Fermentation and cell harvest

All fermentations were performed at 28°C and 180 rpm in baffled shaker flasks filled with 1/10 of their volume in medium. For each strain, three independent pre-cultures were grown overnight in BMG1 (1.34% (w/v) YNB, 1% (w/v) glucose, 4 × 10 − 5% (w/v) biotin, 200 mM potassium phosphate buffer pH 6.0) from single colonies. The cells were spun down and used to inoculate BMG1 medium to an optical density (OD_600_) of 0.5 for glucose batch fermentations that lasted 48 h. For the induction phase, the cells were pelleted, adjusted to an OD_600_ = 10 ± 0.4 in BMM1, and grown for 96 h, while the medium was supplemented with the same amount of methanol (0.5% of the total start volume) every twelve hours. Strains BSY3G-gSec and BSY3G-ΔgSec were also incubated at 20°C for the induction phase under otherwise identical conditions. For harvesting, the cells suspended in BMM1 were mixed 1:1 with ice-cold 5% phenol in absolute ethanol directly after harvesting to quench metabolic activity for subsequent RT-qPCR experiments [88].

### Total RNA extraction and RT-qPCR

To ensure data transparency, the MIQE guidelines [89] were followed (supplementary material 3). Samples were pelleted and concentrated to an OD_600_ = 50 in PBS (0.8% (w/v) NaCl, 0.02% (w/v) KCl, 0.144% (w/v) Na_2_HPO_4_, 0.0245% (w/v) KH_2_PO_4_, pH 7.4) with 2% (v/v) ß-mercaptoethanol and then mechanically lysed (glass beads ø = 0.5 mm, 5 min at 30 Hz) with a Qiagen TissueLyser II (Qiagen N.V., Netherlands). Total RNA was extracted via the Roboklon Universal RNA Kit according to the manufacturer’s instructions (Roboklon GmbH, Germany); RNA extracts were stored for two weeks at −80°C in nuclease-free water. RT-qPCR was carried out using the Luna^®^ Universal One-Step RT-qPCR Kit (New England Biolabs GmbH, Germany) on a Roche LightCycler 480 II (F. Hoffmann-La Roche Ltd., Switzerland). Amplification and melting curve raw data were analyzed with the RDML-tools suite (https://www.gear-genomics.com) [90]. The genes ACT1, ALG9, ENO1, TAF10, TDH3, TFC1, TPI1, PDA1, and PFK1 (primers in supplementary material 4) were investigated as putative reference genes in the samples *K. phaffii* ΔgSec 20°C and gSec 28°C with the geNorm algorithm [54]. The geometric mean of the two most stable reference genes was used to calculate the relative gene expression in every biological replicate (RDML-files in supplementary material 5). Sample No. 2 of *K. phaffii* gSec at 28°C was excluded due to its aberrant growth behavior. The relative expression for all biological replicates was averaged, and subsequently, the gene of interest expression in 75k γ-secalin-producing strains was assessed relative to the null mutant ΔgSec. Error propagation was conducted with the standard deviation of the samples from which the standard error was calculated. For the graphical report, changes in expression were transformed to log2-fold. Significance testing was performed using Student’s t test under the assumption of a normal distribution, with H_0_ stating that there is no difference in the gene of interest-expression between the 75k γ-secalin producing strains and the 75k γ-secalin deletion strain.

### SDS-PAGE and immunoblotting

After mechanical lysis (lysis buffer: 0.137 M NaCl, 1 mM PMSF, 0.1 M EDTA), *K. phaffii* cells were extracted for 60 min at 60°C with 55% (v/v) propan-2-ol, 45% (v/v) lysis buffer with 6% (w/v) DTT [91]. The supernatant was taken up in an equal amount of 2 x Laemmli SDS-PAGE sample buffer. Aliquots corresponding to 5 × 10^7^ cells were then run on a hand-cast 10% Tris-Glycine SDS-PAGE gel and blotted onto a nitrocellulose membrane. After blocking (5% (w/v) milk powder in tris buffered saline (TBS), 150 mM NaCl, 50 mM Tris-HCl, pH 7.4), the membrane was incubated overnight at 8°C either with a horseradish peroxidase (HRP) coupled anti-gliadin antibody (A1052, Sigma-Aldrich Inc., USA, dilution 1:1.000), or with anti-ubiquitin antibody (PA1-187, Thermo Fisher Scientific Inc., USA, dilution 1:1.000); the latter followed by incubation with an HRP-coupled goat anti-rabbit antibody (A0545, Sigma-Aldrich Inc., USA, dilution 1:40.000). After washing with TBS-T (TBS with 0.03% (v/v) tween 20) and TBS, developer solution (TBS, 0.3 mg/mL 4-Chloro-1-Naphtol solution, 0.3% (v/v) H_2_O_2_) was applied at room temperature until bands became visible. The band size was estimated via ImageJ [92].

### Proteomics analysis using liquid chromatography-based tandem mass spectrometry (LC-MS/MS)

A 10% SDS-PAGE gel loaded with cell extracts from *K. phaffii* gSec (three technical replicates) and *K. phaffii* ΔgSec (one technical replicate) were run as described above and stained with Coomassie Blue. Bands corresponding to the marker size of 72 kDa were excised, destained (50% 5 mM triethylammonium bicarbonate (TEAB), 50% ethanol) and dried with ethanol. Cysteine residues were reduced (10 mM dithiothreitol) and alkylated (55 mM chloroacetamide). The gel pieces were further washed (5 mM TEAB) and dehydrated with ethanol. Proteins were overnight in-gel digested with 40 µl chymotrypsin (10 ng/µl, Promega GmbH, Germany) at 37°C. Digestion was stopped by adding 5 µL 5% (v/v) formic acid. The formed peptides were consecutively retained and dried in a centrifugal evaporator (Centrivap Cold Trap − 50, Labconco, US). The samples were freshly resuspended before MS measurements in 12 µL 2% acetonitrile and 0.1% FA, from which 5 µL were injected into the mass spectrometer per measurement.

LC-MS/MS data acquisition was carried out on a Dionex Ultimate 3000 RSLCnano system coupled to an Orbitrap Fusion LUMOS mass spectrometer (ThermoFisher Scientific, USA). Injected peptides were delivered to a trap column (ReproSil-pur C18-AQ, 5 μm, Dr. Maisch, 20 mm × 75 μm, self-packed) at a flow rate of 5 µL/min in 0.1% formic acid in HPLC grade water. After 10 min of loading, peptides were transferred to an analytical column (ReproSil Gold C18-AQ, 3 μm, Dr. Maisch, 450 mm × 75 μm, self-packed) for one minute, and then separated using a 50 min gradient from 4 to 32% of solvent B (0.1% FA, 5% DMSO in acetonitrile) in solvent A (0.1% FA, 5% DMSO in HPLC grade water) at a 300 nL/min flow rate. The Orbitrap Fusion LUMOS mass spectrometer was operated in data-dependent acquisition (DDA) and positive ionization mode. MS1 spectra (360–1300 m/z) were recorded at a resolution of 60k using a normalized automatic gain control (AGC) target value of 100% and a maximum injection time (maxIT) of 50 msec. A cycle time of 2 s was set. Only precursors with charge states 2 to 6 were selected, and dynamic exclusion of 30 s was enabled. Peptide fragmentation was performed using higher energy collision-induced dissociation (HCD) and a normalized collision energy (NCE) of 30%. The precursor isolation window width was set to 1.3 m/z. MS2 resolution was 15.000 with a normalized automatic gain control (AGC) target value of 150% and maximum injection time (maxIT) of 22 msec.

Peptide identification was performed via the MaxQuant software (version 1.6.3.4 [93]) with its built-in search engine Andromeda [94]. MS2 spectra were searched against the UniProt reference proteome *Komagataella phaffii* (UP000000314, 5073 protein entries, downloaded December 2024), supplemented with the amino acid sequence of 75k γ-secalin (Sect. 2) from rye as well as contaminants (built‐in option in MaxQuant). Chymotrypsin + was specified as proteolytic enzyme. Carbamidomethylated cysteine was set as fixed modification. Oxidation of methionine and acetylation at the protein N‐terminus was specified as variable modifications. Results were adjusted to a 1% false discovery rate on the peptide spectrum match (PSM) level and protein level, employing a target‐decoy approach using reversed protein sequences. The minimal peptide length was defined as 7 amino acids, and the “match-between-runs” functionality was disabled.

### ELISA

For the determination of intracellular 75k γ-secalin, aliquots of propanol-extracts corresponding to 2 × 10^6^ cells were quantified by competitive ELISA (RIDASCREEN^®^ Gliadin competitive, R-Biopharm AG, Germany) following the manufacturer’s protocol, omitting the extraction step described therein. Plate reads were processed with RIDASOFT^®^ Win.NET Food & Feed (Art. Nr. Z9996FF, R-Biopharm AG, Germany), the results were calculated to correspond to 5 × 10^7^ cells.

## Supplementary Information

Supplementary Material 1: geNorm analysis of reference genes from *K. phaffii* ΔgSec at 20°C and *K. phaffii* gSec at 28°C, 96 h post-induction.** A** genes with lower geNorm M values are considered more stable, with the most stable genes being ACT1 and TFC1.** B** The number of most stable genes used in the experiment was determined by a geNorm V value below 0.15, leading to the application of two reference genes in this study.

Supplementary Material 2: PCR primer sequences for strain construction. The primers were used for amplification of the *Secale cereale Sec2* gene, the expression cassette BSY3Z, and mutations thereof containing the secretion signals MATα-prepro (S1), OST1-pre (S3), OST1-pre-MATα-pro (S4), or the null mutant.

Supplementary Material 3: MIQE checklist.

Supplementary Material 4: PCR primer sequences for RT-qPCR analysis. The target sequences are divided into reference genes (REF) and genes of interest (GOI).

Supplementary Material 5: RDML-files from RT-qPCR experiments.

## Data Availability

All relevant ELISA, immunoblot, and RT-qPCR data generated or analysed during this study are included in this published article (and its supplementary information files).The mass spectrometric raw files as well as the MaxQuant output files have been deposited to the ProteomeXchange Consortium via the PRIDE partner repository and can be accessed using the identifier PXD062119 (https://proteomecentral.proteomexchange.org/cgi/GetDataset? ID=PXD062119, reviewer account username: reviewer_pxd062119@ebi.ac.uk, password: YXSmElxa9GdH).
