# Supplementary material for: Higher induction temperatures and the native secretion signal peptide promote rye prolamin 75k γ-secalin production in Komagataella phaffii
Source: Microb Cell Fact. 2025 Aug 14;24:185. doi: 10.1186/s12934-025-02809-7 (PMC12351904; doi:10.1186/s12934-025-02809-7)
Supplement: Supplementary file 2 — Supplementary Material 2: PCR primer sequences for strain construction. The primers were used for amplification of the Secale cereale Sec2 gene, the expression cassette BSY3Z, and mutations thereof containing the secretion signals MATα-prepro (S1), OST1-pre (S3), OST1-pre-MATα-pro (S4), or the null mutant. [file 12934_2025_2809_MOESM2_ESM.pdf]

Table 1

Primer name	Sequence 5’-3'
D-gSEC-FWD	CTCTAGTCAAGAGCCAAGTACAATCCCACAAC
D-gSEC-REV	GATTGTACTTGGCTCTTGACTAGAGCAAGTGTTATGG
gSEC-FWD	ATGAAGACCTTACTCATGCTTGC
gSEC-REV	TTAGTGGCCAACAATACCAGTG
KanMX-FWD	GTAAGGAGGACTAAACCATGAGCCATATTCAACGGGAAAC
KanMX-REV	CGTAAGGTGTCAATTTTAGAAAAACTCATCGAGCATC
KanMX-BSY-REV	GAATATGGCTCATGGTTTAGTCCTCCTTACACCTTG
KanMX-BSY-FWD	GAGTTTTTCTAAAATTGACACCTTACGATTATTTAG
mature_gSEC-FWD	AACATGCAAGTCAACCCTAGT
pBSYleft-FWD	AAATTAATCGAACTCCGAATGCGGTTC
pBSYleft-REV	ATTGCAAGCATGAGTAAGGTCTTCATTTTAATTGTAAGTCTTGACTAGAGCAAG
pBSYright-FWD	TCACTGGTATTGTTGGCCACTAAGCGGCCGCTCAAGAG
pBSYright-REV	AAATACATGTGAGCAAAAGGCCAGCAAAAGG
pBSYseq2-FWD	GATAGCCTCTGTTGCTCATC
pBSYseq-REV	ATGGCATTCTGACATCCTCTTG
proMATa-gSEC-REV	CCACTAGGGTTGACTTGCATGTTAGCTTCGGCCTCTCTCTTC
preOST1-gSEC-REV	CCACTAGGGTTGACTTGCATGTTAGCAGAAGACACGTTGAAAAAACATAGG
