# Supplementary material for: Higher induction temperatures and the native secretion signal peptide promote rye prolamin 75k γ-secalin production in Komagataella phaffii
Source: Microb Cell Fact. 2025 Aug 14;24:185. doi: 10.1186/s12934-025-02809-7 (PMC12351904; doi:10.1186/s12934-025-02809-7)
Supplement: Supplementary file 3 — Supplementary Material 3: MIQE checklist. [file 12934_2025_2809_MOESM3_ESM.pdf]

Higher Induction Temperatures and the Native Secretion Signal Peptide Promote the Production of Rye Prolamin 75k γ-Secalin in *Komagataella phaffii*

Kai Büchner[1], Christina Ludwig[2], Roland Kerpes[1]* and Thomas Becker[1]

MIQE checklist

ITEM TO CHECK	IMPORTANCE	CHECKLIST
**EXPERIMENTAL DESIGN**		
Definition of experimental and control groups	**Expected (E)**	Experimental group: *K. phaffii* expressing 75k γ-secalin and signal sequence variants thereof. Control group: null mutant
Number within each group	**E**	3 Biological replicates
Assay carried out by core lab or investigator's lab?	Desired (D)	Investigators lab
Acknowledgement of authors' contributions	D	Included in the manuscript
**SAMPLE**		
Description	**E**	*Komagataella phaffii* sampled from shaking flask cultures, 96 h post methanol induction
Volume/mass of sample processed	D	3 mL sample vol, OD_{600}= 18.0 – 21.0; normalised to OD_{600}= 50 for lysis and OD_{600}= 0.8 for RNA extraction
Microdissection or macrodissection	**E**	not applicable
Processing procedure	**E**	Quenching in Phenol solution with subsequent freezing
If fixed - with what, how quickly?	**E**	5 % Phenol in absolute Ethanol (-20°C) mixed 1:1 with liquid sample directly after sampling
If frozen - how and how quickly?	**E**	directly after Phenol quenching at -80°C
Sample storage conditions and duration (especially for FFPE samples)	**E**	-80°C for 5 days in medium : 5% vol. phenol in abs. Ethanol 1:1, as sampled before
**NUCLEIC ACID EXTRACTION**		
Procedure and/or instrumentation	**E**	Glass bead cell lysis with subsequent spin column RNA extraction
Name of kit and details of any modifications	**E**	Roboklon Universal RNA Kit, Roboklon GmbH, Germany; with glass bead lysis and DNase I digest
Source of additional reagents used	D	VWR International GmbH
Details of DNase or RNAse treatment	**E**	DNase I treatment for 10 min, as per protocol
Contamination assessment (DNA or RNA)	**E**	Denaturing RNA gel electrophoresis for degradation, no-reverse-transcription control in the qPCR
Nucleic acid quantification	**E**	Sheet_3
Instrument and method	**E**	Nano drop 2000c spectrophotometer
Purity (A260/A280)	D	
Yield	D	
RNA integrity method/instrument	**E**	MOPS-Gel electrophoresis
RIN/RQI or Cq of 3' and 5' transcripts	**E**	not applicable
Electrophoresis traces	D	
Inhibition testing (Cq dilutions, spike or other)	**E**	not applicable due to small sample volume
**REVERSE TRANSCRIPTION**		
Complete reaction conditions	**E**	Luna® Universal One-Step RT-qPCR Kit, New England Biolabs, USA
Amount of RNA and reaction volume	**E**	0.1 µg total RNA, 15 µL total reaction volume
Priming oligonucleotide (if using GSP) and concentration	**E**	Individual primers for the genes of interes, 0.4 µM each
Reverse transcriptase and concentration	**E**	Mastermix, according to protocol
Temperature and time	**E**	55°C, 10 min
Manufacturer of reagents and catalogue numbers	D	New England Biolabs, USA
Cqs with and without RT	D*	not applicable
Storage conditions of cDNA	D	not applicable
**qPCR TARGET INFORMATION**		
If multiplex, efficiency and LOD of each assay.	**E**	not applicable
Sequence accession number	**E**	Sheet_2
Location of amplicon	D	
Amplicon length	**E**	Sheet_2
In silico specificity screen (BLAST, etc)	**E**	Primer BLAST, https://www.ncbi.nlm.nih.gov/tools/primer-blast/
Pseudogenes, retropseudogenes or other homologs?	D	not applicable, HAC1 splice variants are targeted
Sequence alignment	D	
Secondary structure analysis of amplicon	D	
Location of each primer by exon or intron (if applicable)	**E**	exons targeted; except for $HAC1^{u}$, where the exon/intron junction was targeted
What splice variants are targeted?	**E**	$HAC1^{u}$, $HAC1^{i}$
**qPCR OLIGONUCLEOTIDES**		
Primer sequences	**E**	Sheet_2
RTPrimerDB Identification Number	D	not applicable
Probe sequences	D**	
Location and identity of any modifications	**E**	not applicable
Manufacturer of oligonucleotides	D	TIB Molbiol Syntheselabor GmbH
Purification method	D	Primer HPLC
**qPCR PROTOCOL**		
Complete reaction conditions	**E**	Luna® Universal One-Step RT-qPCR Kit, New England Biolabs, USA
Reaction volume and amount of cDNA/DNA	**E**	15 µL
Primer, (probe), Mg++ and dNTP concentrations	**E**	Primer conc. 400 nM each
Polymerase identity and concentration	**E**	Manufacturer Master Mix
Buffer/kit identity and manufacturer	**E**	Luna® Universal One-Step RT-qPCR Kit, New England Biolabs, USA
Exact chemical constitution of the buffer	D	
Additives (SYBR Green I, DMSO, etc.)	**E**	Proprietary dye, SYBR green scan mode applied
Manufacturer of plates/tubes and catalog number	D	Azenta, USA; Cat. N° 4TI-0955/0560
Complete thermocycling parameters	**E**	Initial denaturation: 1 min, 95°C; 40x [10 sec, 95°C; 30 sec, 60°C]
Reaction setup (manual/robotic)	D	manual
Manufacturer of qPCR instrument	**E**	Roche LightCycler 480 II
**qPCR VALIDATION**		
Evidence of optimisation (from gradients)	D	
Specificity (gel, sequence, melt, or digest)	**E**	Melting curve, single peaks for all reactions; agarose gel
For SYBR Green I, Cq of the NTC	**E**	none
Standard curves with slope and y-intercept	**E**	not applicable, calculation of individual per-plate efficiencies
PCR efficiency calculated from slope	**E**	not applicable, calculation of individual per-plate efficiencies
Confidence interval for PCR efficiency or standard error	D	
r2 of standard curve	**E**	not applicable
Linear dynamic range	**E**	not applicable
Cq variation at lower limit	**E**	not applicable
Confidence intervals throughout range	D	
Evidence for limit of detection	**E**	not applicable
If multiplex, efficiency and LOD of each assay.	**E**	not applicable
**DATA ANALYSIS**		
qPCR analysis program (source, version)	**E**	LightCycler 480 Software release 1.5.0; RDML tools
Cq method determination	**E**	Proprietary Roche LighCycler
Outlier identification and disposition	**E**	according to RDML tools
Results of NTCs	**E**	none
Justification of number and choice of reference genes	**E**	according to geNorm V and geNorm M
Description of normalisation method	**E**	Modified Pfaffl Method according to Hellemans et al., RDML-Analyze
Number and concordance of biological replicates	D	
Number and stage (RT or qPCR) of technical replicates	**E**	technical duplicates in the qPCR
Repeatability (intra-assay variation)	**E**	not applicable
Reproducibility (inter-assay variation, %CV)	D	
Power analysis	D	
Statistical methods for result significance	**E**	One-way Anova
Software (source, version)	**E**	RDML-Analyze, Libre Office 24.8.5.2
Cq or raw data submission using RDML	D	Supplementary files
