# Supplementary material for: Higher induction temperatures and the native secretion signal peptide promote rye prolamin 75k γ-secalin production in Komagataella phaffii
Source: Microb Cell Fact. 2025 Aug 14;24:185. doi: 10.1186/s12934-025-02809-7 (PMC12351904; doi:10.1186/s12934-025-02809-7)
Supplement: Supplementary file 4 — Supplementary Material 4: PCR primer sequences for RT-qPCR analysis. The target sequences are divided into reference genes (REF) and genes of interest (GOI). [file 12934_2025_2809_MOESM4_ESM.pdf]

| Primer name | Sequence 5’-3' | Target type |
|---|---|---|
| ACT1-FWD | AGGCTTCTGGTATCGACCAAAC | REF |
| ACT1-REV | AGTAGTACCACCGGACATAACG | REF |
| ALG9-FWD | TGGACCAGTTTGCTTCAGATACTC | REF |
| ALG-REV | CGTAGACGCATAGAAGGAGGTAG | REF |
| ENO1-FWD | CCGAATCTATCAAGGCTGCTAC | REF |
| ENO1-REV | GCAATGGTGGTGTCTTCAGTC | REF |
| TAF10-FWD | ACAACCTCAAACGGGAGCTTC | REF |
| TAF10-REV | TAGTGCCCGTACTTGTTCCTC | REF |
| TDH3-FWD | CGACGCTTCTGCCGGTATTC | REF |
| TDH3-REV | TCGACGACTCTGGTGGAGTAAC | REF |
| TFC1-FWD | TGGTGATACCTTCCCATCAGTTG | REF |
| TFC1-REV | CCATAACGCTCAGAAGGCTTCTC | REF |
| TPI1-FWD | TCACCGAGAACAAGCAGAAG | REF |
| TPI1-REV | TAGGCACCAGAAGCTTTGTC | REF |
| PDA1-FWD | ACAGGTATGGTGGACACTCTATG | REF |
| PDA1-REV | AAGCCTGCAATTGGGTCATTTC | REF |
| PFK1-FWD | TTTGGGCAGGAAGCAAGACC | REF |
| PFK1-REV | AGATGCTGAGTCCTGCGAATG | REF |
| DOA10-FWD | CTGGGACGACATTGGAGGTTTAG | REF |
| DOA10-REV | GAGACAGACCAAACTTGGCAAAC | GOI |
| HAC1i-FWD | CGACTACATTACTACAGCTCCATCAG | GOI |
| HAC1i-REV | ATGGTGCTGCTGGATGATGC | GOI |
| HAC1u-FWD | CCAGCAGTGATGACGGATTC | GOI |
| HAC1u-REV | TGTCAACTGATATGTGCCAACTG | GOI |
| HRD1-FWD | TGCCACATGCACCAGTTAACAG | GOI |
| HRD1-REV | GTGAGAGCTTGGTACTCTAGTG | GOI |
